# Prevalence of Fungal and Bacterial Co-Infection in Pulmonary Fungal Infections: A Metagenomic Next Generation Sequencing-Based Study

**DOI:** 10.3389/fcimb.2021.749905

**Published:** 2021-11-01

**Authors:** Zhan Zhao, Junxiu Song, Changqing Yang, Lei Yang, Jie Chen, Xinhui Li, Yubao Wang, Jing Feng

**Affiliations:** ^1^ Respiratory Department, Tianjin Medical University General Hospital, Tianjin, China; ^2^ Institute of Infectious Diseases, The Second Hospital of Tianjin Medical University, Tianjin, China; ^3^ Guangzhou Sagene Biotechnology Company, Limited, Guangzhou, China

**Keywords:** fungal infection, fungal and bacterial co-infection, risk factor, mNGS, antibacterial treatment

## Abstract

With the widespread use of antibacterial drugs and increasing number of immunocompromised patients, pulmonary fungal infections are becoming more common. However, the incidence of pulmonary fungal and bacterial co-infection is rarely reported. In this study, 119 patients definitively diagnosed with pulmonary fungal infections between July 2018 and March 2020 were assessed using metagenomic next-generation sequencing (mNGS) as well as traditional pathogen detection to gauge the incidence of fungal and bacterial co-infection and evaluate the associated risk factors. We found that of the 119 patients with fungal infections, 48 (40.3%) had pulmonary fungal and bacterial co-infection. We identified immunocompromised status and the presence of one or more pulmonary cavities as risk factors associated with fungal and bacterial co-infection. The most commonly isolated fungi species were *Aspergillus, Pneumocystis*, and *Rhizopus.* The most commonly isolated bacterial species were *Pseudomonas aeruginosa, Acinetobacter baumannii*, and *Stenotrophomonas maltophilia.* Seventy-nine (66.4%) patients had received empirical antibiotic treatment before their pathogenic test results became available, and 41.7% (fungal infection group) and 38.7% (fungal and bacterial co-infection group) of the patients had their antibacterial drug dosage changed accordingly. This mNGS-based study showed that the incidence of fungal and bacterial co-infection is significant. Our research outcomes can, thus, guide the use of antibacterial drugs in the treatment of clinical fungal infections.

## Introduction

The human respiratory tract is exposed to various microorganisms present in the ambient atmosphere including fungi. In most individuals, immune function can prevent fungal growth and invasion, but a growing number of immunocompromised patients and the widespread use of antibiotics has led to an increase in the incidence of pulmonary fungal infections (Limper et al., 2011; Denning and Chakrabarti, 2017; Li et al., 2021). Generally, fungal infections are more common in the elderly. However, one report provided evidence that the incidence of fungal infections in hospitalized patients aged 14–30 years has shown a clear upward trend from 2013 to 2019 (Li et al., 2021). Invasive fungal infections are associated with high mortality, with mortality rates as high as 67% being reported among patients with acute infections (Chen et al., 2001). Clearly, pulmonary fungal infections represent a significant clinical and financial burden to the medical community.

To date, few studies have reported the incidence of pulmonary co-infection of fungi and bacterium or the species of co-infecting bacteria. One report described fungal and bacterial co-infection in burn wounds (de Macedo and Santos, 2005). Presently, the clinical treatment for pulmonary fungal infections usually consists of combining antifungal and antibacterial drugs (Guan et al., 2020). In the absence of a complete understanding of fungal and bacterial co-infection, empirical addition of antibacterial drugs could enhance the risk of drug resistance. Furthermore, the non-specific symptoms of pulmonary co-infections, the limitation of diagnostic methods, and the long, time-consuming traditional detection methods with poor detection rates have hampered efforts to distinguish fungal and bacterial co-infection from other pathogenic infections.

Given the limitations of current pathogenic diagnosis, a rapid and accurate alternative method is imperative. Metagenomic next-generation sequencing (mNGS), also known as high-throughput sequencing, is a promising technique that can be used for rapid identification of infectious pathogens without the need for culture, and with greater sensitivity than traditional culture methods (Gu et al., 2019; Wang et al., 2019). Moreover, mNGS furthers the concept of “precision diagnosis and treatment,” as it can be used for multidisciplinary infection diagnosis. According to one report on peripheral pulmonary infectious lesions, the pathogen detection rate *via* mNGS is nearly 89%, which is significantly higher than that *via* traditional detection methods (Huang et al., 2020). Identifying pathogens at an early stage may change the outcome of the disease, but traditional methods based on microbiological and biochemical features are time-consuming and have low detection rates (Petrucelli et al., 2020). Therefore, herein, we used mNGS technology to identify pathogens from lung biopsy samples of patients with fungal infections, or bacterial and fungal co-infections, comparing results to those obtained by traditional methods, and assessing the type and prevalence of the species found to better understand and treat these infections.

## Materials and Methods

### Patients

We analyzed 119 patients with fungal infections, who were admitted to the Respiratory Department of Tianjin Medical University General Hospital from July 2018 to July 2020. All enrolled patient samples were analyzed using mNGS as well as traditional pathogen detection methods. We ascertained the underlying disease and confirmed the heart and coagulation function of all patients. Informed consent was obtained from all subjects before performing biopsy surgery. Immunocompromised patients (those with hematological malignancies, autoimmune diseases, or taking immunosuppressants) were identified. CT scans were performed on all patients using a procedure in keeping with the technical principle of CT scans. Meanwhile, we noted the patient’s anti-fungal or antibiotic treatment for the month prior to receiving the pathogen test. Enrolled patients also underwent relevant examinations according to the condition of the disease, such as galactomannan test, (1,3)-β-D-glucan test, sputum smear microscopy, and culture-based analysis. For traditional pathogen detection methods, normal flora of the skin or respiratory tract was not interpreted as pathogens. All procedures were carried out by trained personnel and the study was approved by the Ethics Review Committee of Tianjin Medical University General Hospital. The study was conducted in accordance with the guidelines outlined in the Declaration of Helsinki.

### Specimen Collection and Processing

Specimens were collected by the trained staff at the Respiratory Endoscopy Center according to standard procedures. The virtual bronchoscope navigation system, endobronchial ultrasound system, and CT imaging were used to precisely locate the lesion and the results were further confirmed *via* rapid on-site evaluation of cytology (ROSE). Transbronchial lung biopsy (TBLB) and bronchoalveolar lavage fluid (BALF) analysis were performed by routine bronchoscopy or ultrathin bronchoscopy (Olympus, Tokyo, Japan). Six to ten pieces of lung tissue were taken from the diseased area, each piece weighing approximately 4 to 6 g. A portion of each lung biopsy was sent to the histopathology laboratory for processing with hematoxylin and eosin, Ziehl-Neelsen acid-fast, and hexamine silver staining. The rest of the tissue was used for mNGS analysis. The bronchopulmonary specimen from the diseased site was rinsed four times with sterile saline and recycled to obtain BALF. A portion of each specimen was stored at 4°C for mNGS analysis, while the remaining BALF samples were processed within 2 h. In the microbiology laboratory, BALF was used for culture and smear microscopy to identify pathogens and also assessed by the GeneXpert mycobacterium tuberculosis (MTB) and galactomannan (GM) tests.

### Metagenomic Next-Generation Sequencing and Analysis

DNA was extracted from BALF and TBLB tissue samples using the TIANamp Micro DNA Kit (TIANGEN BIOTECH, Beijing, China) according to the manufacturer’s instructions. As previously described, DNA libraries were sequenced on the Beijing Genomics Institute sequencer-100 (Miao et al., 2018). High-quality sequencing data were generated by removing low-quality, adapter contamination, duplicated reads and short (length <35 bp) reads, followed by computational subtraction of human host sequences mapped to the human reference genome (hg19) using Burrows–Wheeler Alignment. The remaining data were classified using four microbial reference genomes consisting of bacteria and fungi (NCBI; ftp://ftp.ncbi.nlm.nih.gov/genomes/). The interpretation criteria used to determine the results of the mNGS test were defined *via* reference to previous studies (Petrucelli et al., 2020). 1) For bacteria and fungi, the relative abundance of was greater than 30% at the genus level excluding *Mycobacterium tuberculosis*; 2) *Mycobacterium tuberculosis* was considered positive, once it is satisfied that at least one read was aligned to the reference genome at species or genus level, and 3) When the pathogen was detected by the traditional detection method and the mNGS reads number was more than 50 at the same time, this pathogen can also be considered to be positively detected (Li et al., 2018).

### Statistical Analyses

We used the *t-*test or Chi-square test to compare differences between fungal infection and fungal and bacterial co-infection and used univariate and multivariate logistic regression models to explore the risk factors associated with co-infections. All statistics were calculated by SPSS 22.0 software and results with *P* < 0.05 were considered statistically significant.

## Results

### Characteristics of Pulmonary Fungal Infection

One hundred and nineteen patients with pulmonary fungal infection in Tianjin Medical University General Hospital underwent imaging examinations, traditional pathogenic examinations, and mNGS. Other examinations were also carried out according to clinical needs, such as Xpert and GM tests of BALF, G test, and blood sample tests, among others. According to etiology, 119 patients were divided into a fungal and bacterial co-infection group (n=48) and a fungal infection group (n=71).

The average age for the patients in the fungal and bacterial co-infection group and the fungal infection group was comparable at 43.04 years and 40.77 years, respectively. In the co-infection and fungal infection groups, males accounted for 66.7% and 56.3% patients, respectively. Factors affecting immunity were heavily implicated in fungal infections. We found that 89.6% (n=43) of patients harboring co-infections were immunocompromised, while just 10.4% (n=5) of patients had normal immune function. By comparison, the proportion of immunocompromised patients in the fungal infection group was 74.6% (n=53), while 25.4% (n=18) patients had normal immune function. Results of CT imaging showed that 22 of 119 (18.5%) patients had cavity lesions in the lungs, of whom 13 were accompanied by fungal and bacterial co-infection and the rest (n=9) had only fungal infection. Among the 119 enrolled patients, 21 cases (17.6%) were detected with viruses, of which 10 cases (20.8%) were from the fungal and bacterial co-infection group, and 11 cases (15.5%) were from the fungal infection group (**Table 1**).

**Table 1:** Baseline characteristics and risk factors of pulmonary co-infection.

	Fungal and bacterial co-infection	Fungal infection	Univariable OR^a^(95% CI^b^)	*P* value	Multivariable OR (95% CI)	*P* value
Age (years)	43.04 ± 18.2	40.77 ± 18.8	–	0.580	–	–
Sex						
Male	32 (66.7%)	40 (56.3%)	1.55 (0.72–3.32)	0.258	–	–
Female	16 (33.3%)	31 (43.7%)				
Immune function						
Normal	5 (10.4%)	18 (25.4%)	2.92 (1.00–8.51)	0.043	3.20 (1.07–9.62)	0.038
Defective	43 (89.6%)	53 (74.6%)				
Antibacterial treatment	31 (64.6%)	48 (67.6%)	0.874 (0.40–1.89)	0.732	–	–
CT image manifestation (Cavity lesions)	13 (27%)	9 (12.7%)	2.56 (0.99–6.59)	0.047	2.81 (1.06–7.49)	0.039
Isolated viruses	10 (20.8%)	11 (15.5%)	–	0.453	–	–
Fungus species						
*Aspergillus*	21 (43.8%)	26 (36.6%)	–	0.591^c^	–	–
*Pneumocystis*	11 (22.9%)	19 (26.8%)				
*Rhizopus*	9 (18.8%)	12 (16.9%)				
*Cryptococcus*	3 (6.3%)	8 (11.3)				
*Lichtheimia*	2 (4.2%)	3 (4.2%)				
*Penicillium*	1 (2%)	2 (2.8%)				
*Mucor*	1 (2%)	0 (0%)				
*Scedosporium*	0 (0%)	1 (1.4%)				

a OR, odds ratio.

b CI, confidence interval.

c Calculated by t-test.

### Risk Factors Associated With Fungal and Bacterial Co-Infection

We assessed the role of age, sex, immune function, antibacterial treatment, and pulmonary cavities in pulmonary fungal and bacterial co-infections by univariate and multivariate logistic regression methods. Immunocompromised status had an odds ratio (OR) of 2.92 (95% CI 1.00-8.51, *P* = 0.043), pulmonary cavity had an OR of 2.56 (95% CI 0.99-6.59, *P* = 0.047), and both were significantly associated with fungal and bacterial co-infections. In contrast, age, sex, and antibacterial treatment did not play significant roles in pulmonary fungal and bacterial co-infections (*P*=0.580, 0.258 and 0.732, respectively), as determined by univariate analysis, and therefore, they were excluded from further analysis. We included 119 patients with complete data for immune function and pulmonary cavity (48 co-infections and 71 fungal infections) in the multivariate logistic regression model. Immunocompromised status (OR = 3.20, 95% CI: 1.07-9.62, *P* = 0.038) and pulmonary cavity (OR = 2.56, 95% CI: 1.06-7.49, *P* = 0.039) were found to be risk factors for fungal and bacterial co-infection (**Table 1**). This further confirmed the previous conclusion that immunocompromised status and the presence of pulmonary cavities are substantially related to co-infection of fungi and bacteria.

### Distribution of Fungal Species

**Figure 1:**
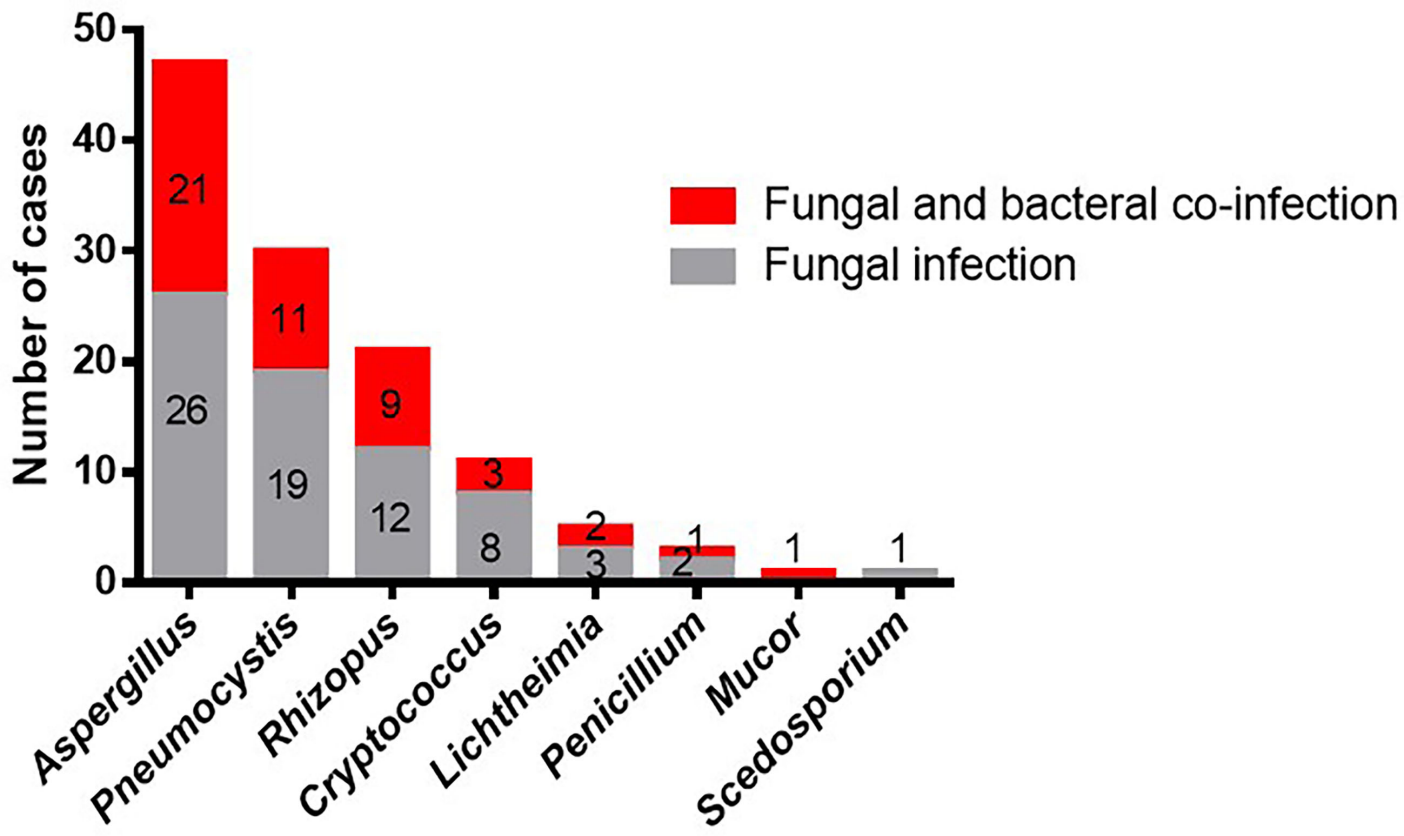
Occurrences of bacterial co-infection with different fungal infections. Red indicates the cases of fungal infections that combine with bacterial infections. Gray indicates the cases of fungal infections. Numbers indicate the number of cases.

In the 119 patients with fungal infections, eight species of fungus were detected by mNGS and conventional laboratory-based diagnostic testing (**Table 1**). The most commonly observed fungal species was *Aspergillus*, which accounted for 47/119 infections (39.5%), followed by *Pneumocystis* (30, 25.2%), and *Rhizopus*, which was observed in 21 cases (17.6%). *Cryptococcus* and *Lichtheimia* were isolated from eleven (9.24%) and five (4.2%) samples, respectively. The relatively rare fungal species observed in this study were *Penicillium, Mucor*, and *Scedosporium* (**Table 1** and **Figure 1**).

The top three fungal species, *Aspergillus, Pneumocystis*, and *Rhizopus*, were most commonly isolated from both the co-infection group and the fungal infection group. However, *Scedosporium* was not observed in the co-infection group, while *Mucor* was not present in the fungal infection group. Moreover, we did not find any difference in the distribution of fungal species between the two groups (*P* > 0.05). In conclusion, *Rhizopus* and *Aspergillus* were the most common species to present with bacterial infections in our study, and the overall occurrence of bacterial infections was unaffected by fungal species.

### Pathogenic Bacterial Species in Patients With Fungal Infections

**Figure 2:**
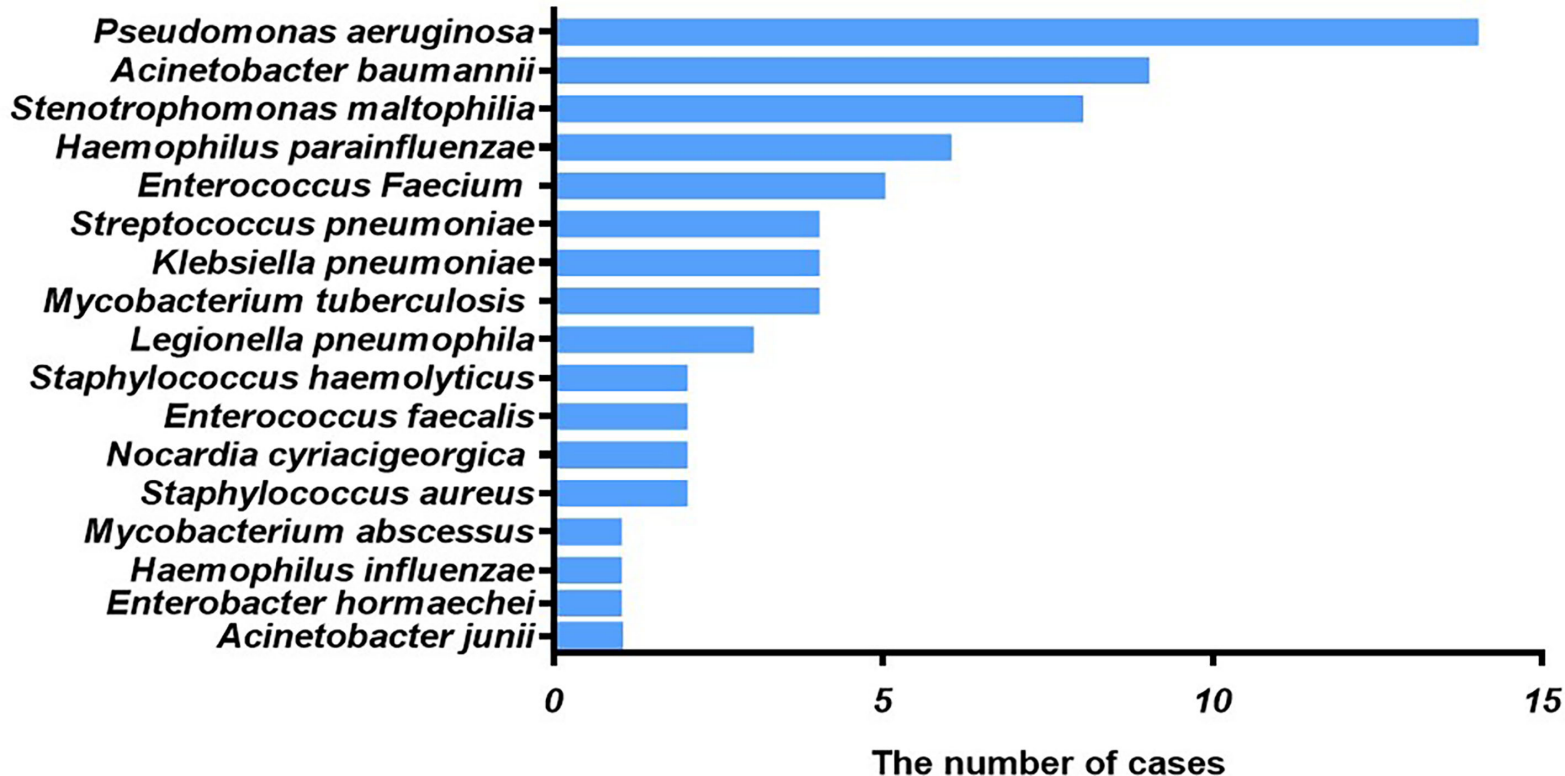
Type of bacteria observed in cases of fungal and bacterial co-infection. The x-axis displays the number of isolates in which the corresponding bacterial species were found.

**Figure 3:**
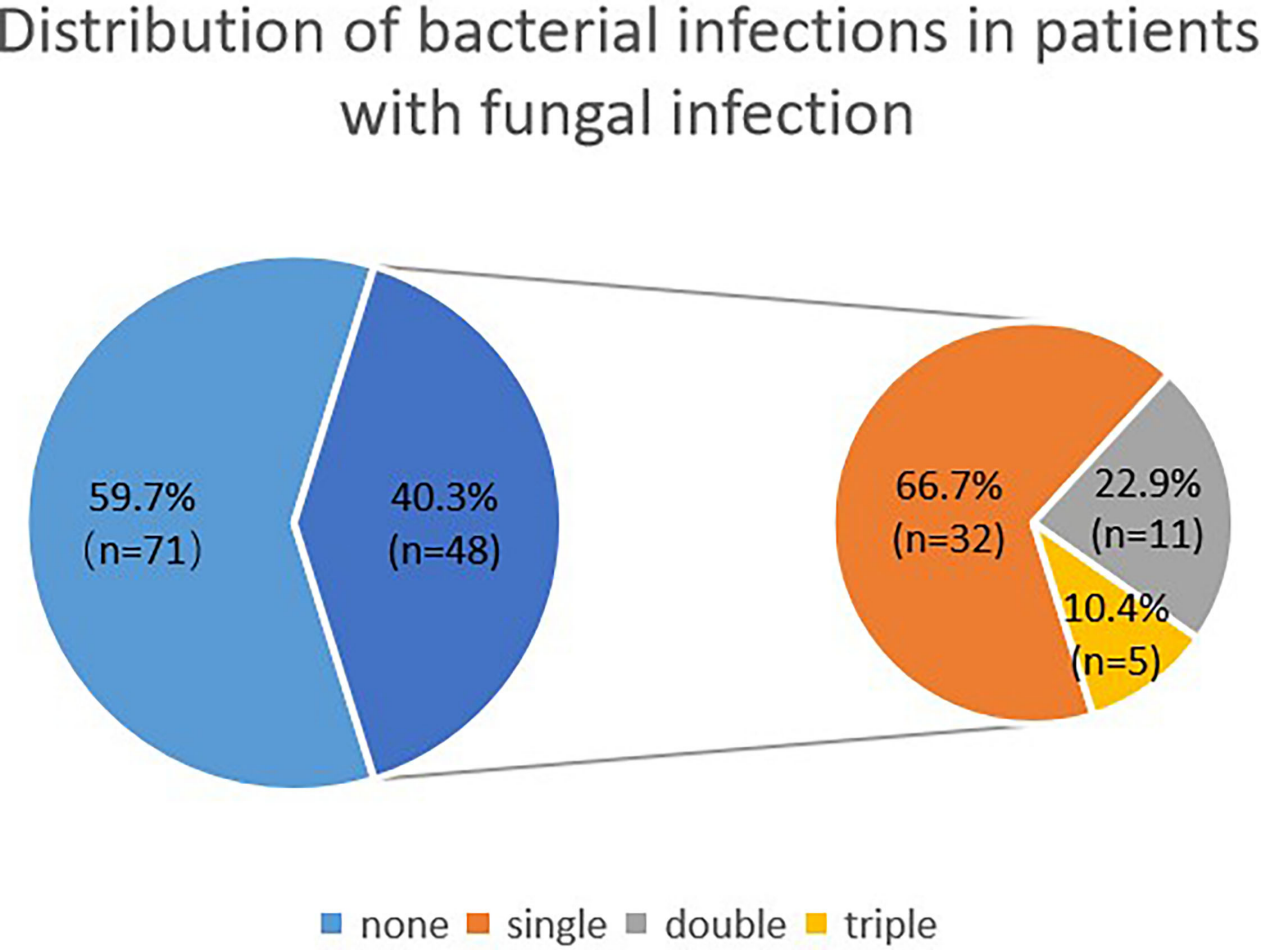
Distribution of bacterial infections in patients with fungal infection. The larger blue pie represents fungal infections *vs* co-infections, and the smaller pie is the distribution of bacterial infections within the co-infection group.

Among patients with fungal and bacterial co-infections, 17 species of bacteria were identified from the 69 strains that were isolated. For the 69 strains of bacteria, the detection rate using mNGS was 89.9%, while that of conventional laboratory-based diagnostic testing was only 21.7% with *P* < 0.05. The most frequently detected bacteria were *Pseudomonas aeruginosa* (n=14) followed by *Acinetobacter baumannii* (n=9) and *Stenotrophomonas maltophilia* (n=8), all belonging to gram-negative bacteria (**Figure 2**). These bacteria are three of the main pathogens often seen in hospital infections (Petrucelli et al., 2020). In addition, *Haemophilus parainfluenzae* (n=6) and *Enterococcus faecium* (n=5) co-occurred with fungi. In this study, *Streptococcus pneumoniae*, *Klebsiella pneumoniae*, and *Mycobacterium tuberculosis* appeared at the same frequency in co-infections (n=4). The strains identified less commonly were *Legionella pneumophila*, *Staphylococcus haemolyticus*, *Enterococcus faecalis*, *Nocardia cyriacigeorgica*, *Staphylococcus aureus, Mycobacterium abscessus, Haemophilus influenzae, Enterobacter hormaechei*, and *Acinetobacter junii.* Overall, the frequency of these bacterial species remained the same as in nosocomial infections, with nonfermenting gram-negative bacilli, followed by enterobacteria. For the fungal and bacterial co-infection group, 66.7% (n=32) of patients infected by fungi were co-infected with a single bacterium, 22.9% (n=11) of patients were co-infected with two kinds of bacteria, and 10.4% (n=5) of patients were co-infected with three kinds of bacteria (**Figure 3**). Which bacterial species were found co-infecting with a particular fungal pathogen was shown in **Supplementary Table 1**.

### Modification of the Treatment Strategy Due to Etiological Results

We Collected The Clinical Treatment Data Of The 119 Cases, Of Which 66.4% (79 cases: 48 from the fungal infection group and 31 from the co-infection group), had received empirical antibiotic treatment before their pathogenic test results became available. Subsequently, the use of antibacterial drugs was appropriately adjusted. The number of patients in the fungal infection group and co-infection group was 14 (29.2%) and 12 (38.7%), respectively. In the fungal infection group, six patients stopped using antibacterial drugs. Treatment adjustments included replacement upgrade and de-escalation of antibiotics. Overall, empirical use of antibacterial drugs was changed in 41.7% of the patients in the fungal infection group and 38.7% of patients in the fungal and bacterial group (**Table 2**).

**Table 2:** The influence of etiology on clinical antibacterial treatment.

	Fungal infection (case)	Fungal and bacterial infection (case)
Treatment before etiology		
Antibacterial drugs	48	31
Adjustment according with etiology		
Adjusted antibacterial drugs	14 (29.2%)	12 (38.7%)
Stopped antibacterial drugs	6 (12.5%)	–

## Discussion

Current evidence on the incidence of pulmonary fungal infections accompanied by other pathogenic infections is sparse, with most studies focusing on the relationship between *Aspergillus* and *Pseudomonas aeruginosa* infection (Briard et al., 2015; Briard et al., 2016). Our results showed that 40.3% of patients with pulmonary fungal infections had fungal and bacterial co-infections. The clinical manifestations of fungal and bacterial co-infections are more serious than those of fungal infections alone. Here, we only studied the co-existence of bacteria and fungi; however, we do not know which microorganism appeared first in the pulmonary infections. Some studies have reported that *A. fumigatus* colonization is preceded by fungal infection in patients with chronic obstructive pulmonary diseases (COPD) and cystic fibrosis (Borman et al., 2010; Gago et al., 2019).

Microorganisms do not exist alone, and invariably exhibit direct or indirect communication among themselves (Kolwijck and van de Veerdonk, 2014). Compared with a single pathogen infection, a differential immune response is mounted when patients are infected with multiple pathogens, and this may lead to different clinical outcomes. At present, relatively few studies have focused on the interaction between fungi and bacteria, especially in the respiratory system. Prior studies were mostly performed *in vitro* and in animal models, and the results typically demonstrated that bacteria had an inhibitory effect on fungi (Ferreira et al., 2015; Sass et al., 2018). For example, in a murine model of pulmonary disease, immunosuppressed mice with *A. fumigatus* and *P. aeruginosa* co-infection had a higher survival rate than animals infected with *A. fumigatus* alone. However, our result shows that the proportion of patients with bacterial-fungal co-infection is as high as 40.3% among patients with pulmonary fungal infections, which may suggest that interspecies microbial interactions have a positive correlation.

In our study, among the eight types of fungus isolated, *Aspergillus*, *Pneumocystis*, and *Rhizopus* were the most prevalent. However, results of another study analyzed the pathogens in 1,644 in-patients with pulmonary fungal infection and found *Aspergillus*, *Cryptococcus*, and *Talaromyces marneffei* as the three most common fungi (Li et al., 2021). Comparing their study with ours, only the *Aspergillus* was commonly observed in patients with bacterial and fungal co-infections, which may be ascribed to the different geographical distribution and the considerably larger number of cases in the earlier study. For example, *Talaromyces* is epidemic in mountainous/tropical regions in south east Asia, which is likely not present in patients accepted at the location examined in this study presuming no such travel history.

Fungal infections commonly occur in immunocompromised individuals. However, in our study, a large proportion (19.3%) of patients with pulmonary fungal infections had normal immune function. An increasing number of reports show that immunocompetent patients suffer from fungal infections, probably due to environmental exposures, genetic factors, or structural pulmonary risk factors (Denning and Chakrabarti, 2017; Latgé and Chamilos, 2019). Immunocompromised status was nevertheless a significant risk factor for co-infection of bacteria and fungi, which is consistent with previous studies (Zhou et al., 2020). The presence of pulmonary cavity lesions also increased susceptibility to co-infection. Pulmonary cavitation is a relatively serious pulmonary affliction, which may cause massive pulmonary hemorrhage and affect the ability of drugs to reach the diseased site, leading to ineffective treatment (AlShanafey et al., 2019). Antibiotic treatment has been shown to lead to fungal dominance (Jain et al., 2021), however, it is not identified as a risk factor in this study, which is likely due to the patient’s short-term treatment. CT cavitary images may be used as the standard for comparing the severity of infection in cases of fungal infection to those with fungal and bacterial co-infection. In our study, the proportion of pulmonary cavitation was significantly higher in the co-infection group than in the fungal infection group. Hence, we concluded that the combined bacterial infection may aggravate the disease, which is consistent with current findings in humans. A retrospective cohort study showed that compared with *P. aeruginosa* infection alone, *A. fumigatus* and *P. aeruginosa* co-infection caused more rapid decline in patients with lung function and worsened clinical outcomes (Amin et al., 2010). Another study suggested that *S. maltophilia* and *P. aeruginosa* infections are associated with a higher probability of concurrent *Aspergillus* infection, which exacerbates clinical manifestations (Granchelli et al., 2018). These findings explain, to some extent, the diversity of interactions and clinical effects observed in fungal and bacterial co-infections. Similarly, the diversity of interactions between microorganisms also partially explains why some clinical treatments against pathogenic bacteria fail. In our study, the appearance of pulmonary cavitation was not common. There are numerous unknown factors surrounding these observations, and further research is required to fully understand the mechanisms at play.

We did not find a difference in the distribution of bacterial species in people with normal and defective immune functions. However, since we only studied the distribution of bacterial species in the short term after the use of antibacterial drugs, we do not know the long-term effects. For fungal and bacterial co-infections, 17 types of bacteria were identified, almost all of which were hospital-associated pathogens. *P. aeruginosa*, the most commonly isolated bacterial species in this study, is a gram-negative opportunistic pathogen existing in diverse environmental settings that harbors multi-drug resistance and poses serious therapeutic challenges (Lister et al., 2009; Leroy et al., 2021). Interestingly, four strains of *Mycobacterium tuberculosis* were isolated in pulmonary bacterial and fungal co-infection, of which three were co-infected with *Aspergillus* and one with *Rhizopus*. A study once reported 8 cases of *Cryptococcus* and *Mycobacterium tuberculosis* co-infections, and most of them occurred in the brain (Chen et al., 2016). Based on statistical analysis and the description of the bacteria likely to co-infect with fungi in patients with pulmonary fungal infection, our findings will be useful for future clinical management of pulmonary fungal diseases.

The identification of co-infection of fungi and bacteria had a notable impact on clinical treatment. Fungal infections are treated with only antifungal treatment, while antibacterial and antifungal treatment is offered when patients are co-infected. In our study, a large proportion (66.4%) of patients had received empirical antibiotic treatment before their pathogenic test results were available. After the etiological results became known, 40.5% of the previously used empirical antibacterial drugs were discontinued or changed, since patients without bacterial co-infection may have other diseases, necessitating the continued use of antibacterial drugs. Anti-fungal or antibiotic treatment can skew the composition of the species that colonize the host, which consequently show less diversity. It is quite common for immunocompromised patients to be prescribed preventive antibiotic use.

mNGS is an unbiased approach for sequence identification of pathogenic microorganisms. One study revealed that mNGS is advantageous for the evaluation of fungal infections and suggested that mNGS combined with smear analyses should be used as a routine diagnostic tool for identifying invasive fungal infections (Li et al., 2018). We used mNGS combined with conventional laboratory-based diagnostic testing to gain more accurate and comprehensive information related to co-infecting microbes in patients with pulmonary fungal infections. mNGS offers the important advantage of detecting infectious pathogens, since it is less affected by prior antibiotic exposure.

## Limitations

Anti-fungal or antibiotic treatment can change the composition and diversity of the microbial species present in the body. As we only obtained each patient’s medication history for one month before diagnosis, we cannot be sure of the impact their earlier medication history might have had on the test results.

## Conclusions

The incidence of fungal and bacterial co-infections is considerable in our mNGS-based study. Our research results should lead to more rational and precise anti-infective treatments, especially for patients who are difficult to diagnose by conventional methods, thereby having a positive impact on assessment of risk and clinical outcomes in these cases.

## Data Availability Statement

The data presented in the study are deposited in the NCBI and EMBL-EBI repository, accession number PRJNA773581 and PRJEB48166, respectively.

## Ethics Statement

The studies involving human participants were reviewed and approved by the Ethics Review Committee of Tianjin Medical University General Hospital. The patients/participants provided their written informed consent to participate in this study.

## Author Contributions

YW and JF contributed to the research design and revision of the manuscript. ZZ drafted the research protocol, analyzed the results, and drafted the manuscript. JS conducted data analysis and assisted in writing the manuscript. CY and LY conducted data acquisition and analysis. JC and XL contributed to the data analysis and data deposition in an acceptable repository. All authors approved the submitted version and agreed to be responsible for all aspects.

## Funding

This research was supported by grants from National Science and Technology Major Project of China (No.2018ZX10305409-001-001), National Natural Science Foundation of China (81970083, 81270144, 81570084 and 30800507 to JF), and the National Key Technology R&D Program, China (2015BAI12B00 to JF).

## Conflict of Interest

Authors JC and XL were employed by company Guangzhou Sagene Biotechnology Company, Limited.

The remaining authors declare that the research was conducted in the absence of any commercial or financial relationships that could be construed as a potential conflict of interest.

## Publisher’s Note

All claims expressed in this article are solely those of the authors and do not necessarily represent those of their affiliated organizations, or those of the publisher, the editors and the reviewers. Any product that may be evaluated in this article, or claim that may be made by its manufacturer, is not guaranteed or endorsed by the publisher.
