# Supplementary material for: Prevalence of Fungal and Bacterial Co-Infection in Pulmonary Fungal Infections: A Metagenomic Next Generation Sequencing-Based Study
Source: Front Cell Infect Microbiol. 2021 Nov 1;11:749905. doi: 10.3389/fcimb.2021.749905 (PMC8591235; doi:10.3389/fcimb.2021.749905)
Supplement: Supplementary file 1 [file Table_1.docx]

Supplementary Table 1. Bacterial species found co-infecting with corresponding fungal pathogen

| Fungus species | Bacterial species | | | | |
| --- | --- | --- | --- | --- | --- |
| *Aspergillus*（21） | *Acinetobacter baumannii*（5） | *Pseudomonas aeruginosa*（4） | *Stenotrophomonas maltophilia*（4） | *Streptococcus pneumoniae*（3） | *Haemophilus parainfluenzae*（3） |
|  | *Mycobacterium tuberculosis* (3) | *Enterococcus*  *faecium* (1) | *Klebsiella pneumoniae* (1) | *Haemophilus influenzae* (1) | *Staphylococcus haemolyticus* (1) |
|  | *Enterococcus Faecium* (1) | *Acinetobacter*  *junii* (1) | *Staphylococcus aureus* (1) |  |  |
| *Pneumocystis* (11) | *Pseudomonas aeruginosa* (5) | *Legionella*  *pneumophila* (2) | *Acinetobacter baumannii* (1) | *Haemophilus  parainfluenza* (1) | *Mycobacterium abscessus* (1) |
|  | *Nocardia cyriacigeorgic* (1) | *Staphylococcus*  *aureus* (1) | *Enterococcus faecalis* (1) | *Stenotrophomons maltophilia* (1) | *Staphylococcus haemolyticus* (1) |
| *Rhizopus* (9) | *Pseudomonas aeruginosa* (2) | *Klebsiella*  *pneumoniae* (2) | *Enterococcus faecium* (2) | *Stenotrophomonas maltophilia*(1) | *Acinetobacter baumannii* (1) |
|  | *Enterobacter hormaechei* (1) | *Haemophilus parainfluenzae* (1) | *Mycobacterium tuberculosis* (1) | *Streptococcus pneumoniae* (1) |  |
| *Cryptococcus* (3) | *Pseudomonas aeruginosa* (2) | *Nocardia*  *cyriacigeorgica* (1) | *Legionella pneumophila* (1) |  |  |
| *Lichtheimia*（2） | *Enterococcus faecium (1)* | *Enterococcus*  *faecalis*（1） | *Acinetobacter baumannii（1）* | *Pseudomonas aeruginosa*（1） | *Stenotrophomonas maltophilia*（1） |
| *Penicillium*（1） | *Acinetobacter baumannii*（1） | *Stenotrophomonas maltophilia*（1） | *Haemophilus parainfluenzae*（1） |  |  |
| *Mucor*（1） | *Klebsiella pneumoniae*（1） |  |  |  |  |
